# Myocardial triglyceride content at 3 T cardiovascular magnetic resonance and left ventricular systolic function: a cross-sectional study in patients hospitalized with acute heart failure

**DOI:** 10.1186/s12968-016-0228-3

**Published:** 2016-02-05

**Authors:** Pen-An Liao, Gigin Lin, Shang-Yueh Tsai, Chao-Hung Wang, Yu-Hsiang Juan, Yu-Ching Lin, Ming-Ting Wu, Lan-Yan Yang, Min-Hui Liu, Tsun-Ching Chang, Yu-Chun Lin, Yu-Chieh Huang, Pei-Ching Huang, Jiun-Jie Wang, Shu-Hang Ng, Koon-Kwan Ng

**Affiliations:** 1 Department of Medical Imaging and Intervention, Chang Gung Memorial Hospital, Linkou and Chang Gung University, 5 Fuhsing Street, Gueishan, Taoyuan, 333 Taiwan; 2 Institute for Radiological Research, Chang Gung University, Taoyuan, Taiwan; 3 Graduate Institute of Applied Physics, National Chengchi University, Taipei, Taiwan; 4 Department of Cardiology and Heart Failure Center, Chang Gung Memorial Hospital, Keelung, Taiwan; 5 Department of Radiology, Chang Gung Memorial Hospital, Keelung and Chang Gung University, Keelung, Taiwan; 6 Department of Radiology, Kaohsiung Veterans General Hospital, Kaohsiung, Taiwan; 7 Clinical Trial Center, Chang Gung Memorial Hospital at Linkou, Taoyuan, Taiwan

**Keywords:** Heart failure, Left ventricular systolic function, Magnetic resonance spectroscopy, Myocardial triglyceride content, Cardiovascular magnetic resonance

## Abstract

**Background:**

Increased myocardial triglyceride (TG) content has been recognized as a risk factor for cardiovascular disease. However, its relation with cardiac function in patients on recovery from acute heart failure (HF) remains unclear. In this cross-sectional study, we sought to investigate the association between myocardial TG content measured on magnetic resonance spectroscopy (^1^H-MRS) and left ventricular (LV) function assessed on cardiovascular magnetic resonance (CMR) in patients who were hospitalized with HF.

**Methods:**

A total of 50 patients who were discharged after hospitalization for acute HF and 21 age- and sex-matched controls were included in the study. Myocardial TG content and LV parameters (function and mass) were measured on a 3.0 T MR scanner. Fatty acid (FA) and unsaturated fatty acid (UFA) content was normalized against water (W) using the LC-Model algorithm. The patient population was dichotomized according to the left ventricular ejection fraction (LVEF, <50 % or ≥ 50 %).

**Results:**

H-MRS data were available for 48 patients and 21 controls. Of the 48 patients, 25 had a LVEF <50 % (mean, 31.2 %), whereas the remaining 23 had a normal LVEF (mean, 60.2 %). Myocardial UFA/W ratio was found to differ significantly in patients with low LVEF, normal LVEF, and controls (0.79 % vs. 0.21 % vs. 0.14 %, respectively, *p* = 0.02). The myocardial UFA/TG ratio was associated with LV mass (*r* = 0.39, *p* < 0.001) and modestly related to LV end-diastolic volume (LVEDV; *r* = 0.24, *p* = 0.039). We also identified negative correlations of the myocardial FA/TG ratio with both LV mass (*r* = -0.39, *p* < 0.001) and LVEDV (*r* = -0.24, *p* = 0.039).

**Conclusions:**

As compared with controls, patients who were discharged after hospitalization for acute HF had increased myocardial UFA content; furthermore, UFA was inversely related with LVEF, LV mass and, to a lesser extent, LVEDV. Our study may stimulate further research on the measure of myocardial UFA content by ^1^H-MRS for outcome prediction.

**Trial registration:**

ClinicalTrial.gov: NCT02378402. Registered 27/02/2015

**Electronic supplementary material:**

The online version of this article (doi:10.1186/s12968-016-0228-3) contains supplementary material, which is available to authorized users.

## Background

Heart failure (HF), a clinical syndrome in which the heart is unable to provide sufficient blood flow to meet metabolic requirements, is a major public health concern because of its high prevalence, poor prognosis, and significant healthcare costs [1]. In Taiwan, cardiovascular-related deaths have increased significantly from 4.3 to 6.5 persons per million population between 1981 and 2009 [2]. Despite major efforts to improve treatment of acute HF in the hospital setting, the 3 month readmission rates remain as high as 25–50 % and the 5 year survival is less than 50 % [1]. Consequently, more effective therapeutic targets and improved management approaches are urgently needed.

Although a beating heart utilizes fatty acids as a primary source of energy [3], an increased level of myocardial triglyceride (TG) content results in lipotoxicity and may contribute to the development of cardiac dysfunction [4–6]. Consequently, the in vivo measurement of myocardial TG content and metabolic activity has been proposed for guiding appropriate treatment in hospitalized HF patients [7].

Proton magnetic resonance spectroscopy (^1^H-MRS) is increasingly being used as a non-invasive, non-radiation exposure technique for the clinical assessment of myocardial lipid content [8]. In recent years, the use of high-field scanners (3.0 T) has improved the chemical shift resolution and signal-to-noise ratio of cardiac ^1^H-MRS, ultimately allowing the discrimination between fatty acids (FA) and unsaturated fatty acids (UFA) deposition in the myocardium. However, studies focusing on the clinical usefulness of ^1^H-MRS imaging for the metabolic evaluation of HF patients are particularly lacking. We therefore designed the current cross-sectional investigation to analyze the associations between myocardial TG content measured on ^1^H-MRS and left ventricular (LV) function assessed on cardiovascular magnetic resonance (CMR) in patients who were hospitalized with HF. We demonstrate that patients who were discharged after hospitalization for acute HF had increased myocardial UFA content, which was inversely related with LV ejection fraction (LVEF), LV mass and, to a lesser extent, LV end-diastolic volume (LVEDV).

## Methods

### Ethics, consent and permissions

This study was approved by our institutional review board (IRB 102-2772A3), and the study participants provided their written informed consent. The study was registered at ClinicalTrials.gov (Identifier: NCT02378402).

### Study design and participants

This study was conducted at a tertiary referral hospital with a dedicated HF center. The inclusion criteria for patients were as follows: (1) having been discharged from the Keelung Chang Gung Memorial Hospital following hospitalization for acute HF in the 6–12 months preceding the study; (2) absence of known coronary artery disease involving the cardiac septum; (3) age between 20 and 70 years; (4) willingness to comply with the standard treatment and follow-up schedule proposed by the HF center; (5) willingness to provide written informed consent. Patients were excluded if they met one of the following criteria: (1) lack of compliance or inability to follow treatment or scheduled visits; (2) contraindications to CMR (e.g., cardiac pacemaker, metal implants, claustrophobia, or inability to cooperate with the examiners); (3) pregnancy or breastfeeding; and (4) positive history of open cardiac surgery. A multidisciplinary team centrally reviewed medical records of eligible patients to ensure the enrollment quality. The day before their CMR, the study participants were requested to fast overnight [9]. The patient population was dichotomized according to the LVEF at the time of CMR, as follows: low LVEF, <50 % and normal LVEF, ≥ 50 %. Age- and sex-matched volunteers without a history of cardiovascular disorders served as controls. The reproducibility between two repeated ^1^H-MRS measures within a single CMR session was assessed in the first 10 control subjects enrolled in the study. Clinical variables and serum lipid parameters (total cholesterol, high-density lipoprotein cholesterol, low-density lipoprotein cholesterol, very low-density lipoprotein cholesterol, and triglycerides) were collected within 1 month of CMR.

### CMR

CMR was performed on a 3.0-Tesla Skyra scanner (Siemens Medical Systems, Erlangen, Germany) operating on the VD13 platform, with an 18-channel phased-array receiver body coil. Short-axis (contiguous 8-mm-thick slices) and standard long-axis views (2-, 3- and 4-chamber views) cine images were obtained using Steady-State Free Precession (SSFP) cine imaging with the following parameters: repetition time, 3.4 ms; echo time, 1.2 ms; matrix, 256 × 256; field of view, 34 to 40 cm.

### ^1^H-MRS

Short-axis and 4-chamber SSFP cine images were obtained to prescribe the locations of MRS voxels with saturation slabs across subcutaneous and pericardial fat. The B0 shimming parameters were optimized with a (breath-hold) rapid B0 mapping method. Localized 1D MRS with Point-Resolved SpectroScopy (PRESS) was performed by selecting a 2 × 2 × 1-cm^3^ spectroscopic volume within the interventricular septum with the following parameters: nominal TR/TE, 550 ms/33 ms; 64 averages; window size, 1024 points; bandwidth, 2000 Hz. Cardiac gating was determined from the manufacturer’s vector ECG signal, with the first pulse of the PRESS localization sequence being around the end-systole phase (before the beginning of the cardiac motion) [9–17]. The trigger delay was optimized on cine images to avoid signal contamination from the blood pool for accomplishing water suppression [18]. Myocardial TG signals and the water signal were acquired from spectra with water suppression and without water suppression, respectively. Patients breathed freely during spectroscopic acquisition. The spectroscopic signals were acquired at end-systole and end-expiration with electro-cardiographic triggering and respiratory navigator gating. Respiratory gating was triggered with a navigator placed near the right dome of the liver close to the heart. We used Prospective Acquisition Correction navigator echoes (PACE) to control for the respiratory movement (within a pre-defined acceptance window of 5 mm around end expiration) and an electrocardiograph-derived R wave to control for cardiac pulsation. The CMRS scan time was approximately 10 min. To assess reproducibility, we measured with 3 T ^1^H-MRS the composition of a phantom consisting of both UFAs and saturated FAs. Clinical MRS measures were repeated in the first ten control subjects within a single CMR session, with spectra reacquired using the same localizations and settings.

### Image analysis

LVEF was calculated from short-axis cine LV images with vendor-provided post-processing software (Argus Viewer and Function, Siemens Medical Solutions, Erlangen, Germany) on a separate workstation. LV endocardial and epicardial borders were traced manually at end-diastole and end-systole on the short-axis cine images to determine the LVEF and end-diastolic LV mass for each slice, and the respective EDV, end-systolic volume (ESV) were obtained from the tracing. The LV global function index (LVGFI) was calculated using the following formula:

$$ \mathrm{LVGFI} = \left[\mathrm{LVSV}/\left(\left(\mathrm{LV}\mathrm{EDV}+\mathrm{LVESV}\right)/2+\left(\mathrm{L}\mathrm{V}\ \mathrm{mass}/1.05\right)\right)\right] \times 100 $$

**Fig. 1:**
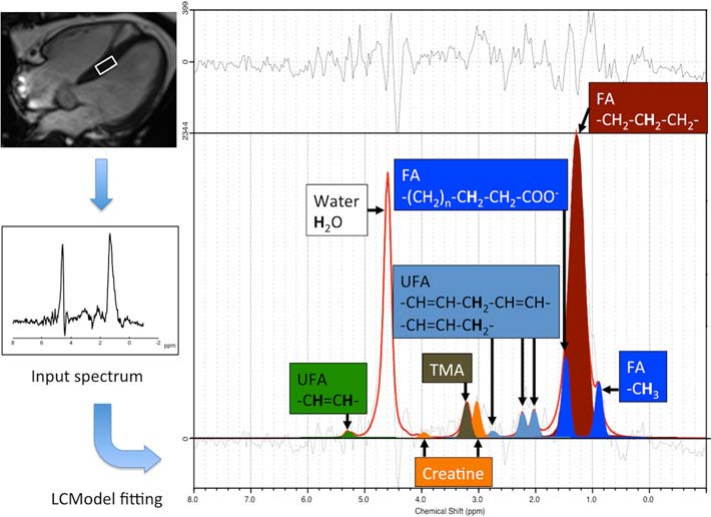
Myocardial CMR spectroscopy. A 2 × 2 × 1-cm^3^ spectroscopic volume was acquired from the interventricular septum during the systolic phase (*left upper inset*) to generate an input spectrum (*left lower inset*). ^1^H-CMR spectra were fitted and analyzed using the LC Model software (Provencher, Ontario, CA, USA). We quantified the total myocardial TG resonance as well as its components – including fatty acids (FA, lipid resonances δ 0.9, 1.3, and 1.6 ppm) and unsaturated fatty acids (UFA, lipid resonance δ 2.1 and 2.3, 2.8, 5.3 ppm). TMA, trimethyl amide

^1^H-MRS spectra were analyzed using the LC Model software (Provencher, Ontario, CA, USA), which automatically calculates a weighted coherent average over the multiple channels and analyzes the resultant spectra (Fig. 1). The metabolites of interest were determined using simulated macromolecule resonances, with their concentrations ranging from 0 to 10 parts per million (ppm). The Cramer-Rao lower bounds (CRLB), which simultaneously account for both resolution and noise, were used as an estimated error in metabolite quantification. All of the spectra in the single scan session were included and averaged, whereas metabolite estimates were excluded from the analysis when the CRLB value exceeded the 50 % range. The variation of the water signal amplitude in the individual spectra acquired without water suppression was calculated. We quantified the total myocardial TG resonance as well as its components including FA (lipid resonances δ 0.9, 1.3 and 1.6 ppm) and UFA (lipid resonance δ 2.1 and 2.3, 2.8, 5.3 ppm) from water-suppressed spectra. We also determined the water resonance (~ δ 4.7 ppm) from spectra without water suppression. Myocardial TG content relative to water as well as relative amounts of myocardial TG was calculated from the available data.

### Statistical analysis

The study variables were summarized using descriptive statistics. A Bland-Altman plot was used to assess the reproducibility between two-repeated ^1^H-MRS measures within a single CMR session in the first ten subjects included in the study [19]. Continuous variables were analyzed with the Mann–Whitney *U* test (two-group comparisons) or one-way analysis of variance (ANOVA, three-group comparisons). Bivariate correlation analyses (Spearman’s rho) were performed to test the association between LV parameters and myocardial TG content. A value of *p* < 0.05 (two-sided) was considered statistically significant.

## Results

### Clinical and biochemical characteristics of the study participants

**Fig. 2:**
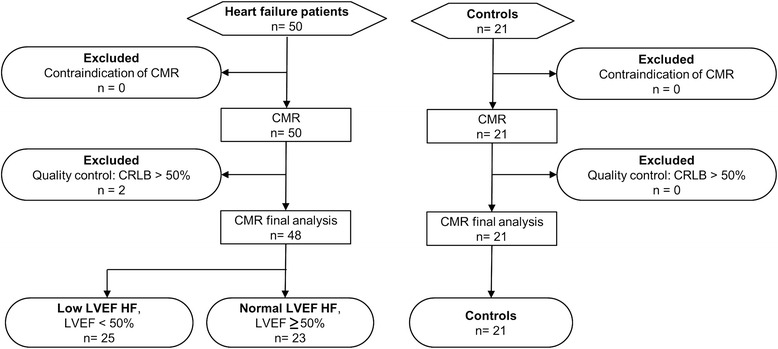
Study flow chart. Summary of the patient selection algorithm and number of subjects in the three study groups: controls, HF (heart failure) patients with low left ventricular ejection fraction (LVEF), and HF patients with normal LVEF

A total of 50 patients who were discharged after hospitalization for acute HF and 21 age- and sex-matched normal controls were included in the study. The flow chart of patient selection algorithm, and the numbers of participants recruited in each subgroup is summarized in Fig. 2. After quality control, satisfactory ^1^H-MRS data of 48 patients and 21 controls were included for analysis. Of the 48 patients, 25 had a low LVEF (mean, 31.2 %), whereas the remaining 23 had a normal LVEF (mean, 60.2 %). The distribution of the clinical characteristics of HF patients with low and normal LVEF was as follows: history of ischemic heart disease, 32 and 35 %, respectively; diabetes mellitus (DM), 58 and 74 %, respectively; dilated cardiomyopathy, 20 and 17 %, respectively; and myocarditis, 12 and 4 %, respectively. HF patients (either with low or normal LVEF) did not differ significantly from controls in terms of age, sex, body weight, and body mass index (BMI; Table 1). The mean diastolic blood pressure was significantly lower in both the low LVEF (74.3 mmHg) and normal LVEF (73.7 mmHg) groups than in controls (83.7 mmHg, *p* = 0.01). Serum HDL levels were significantly higher in HF patients with low LVEF (45.4 mg/dL) compared with the normal LVEF group (38.6 mg/dL, *p* = 0.02).

**Table 1:** Clinical and biochemical characteristics of the study participants

	HF patients with low LVEF	HF patients with normal LVEF	Control subjects	*p* value
	(*n* = 25)	(*n* = 23)	(*n* = 21)	
Clinical profile				
Male sex, n (%)	21 (84 %)	22 (96 %)	17 (81 %)	0.31
Age (years)	56.0 ± 7.7	56.1 ± 8.7	58.9 ± 7.2	0.39
Height (cm)	166.8 ± 8.6	165.8 ± 7.0	164.3 ± 5.0	0.48
Weight (kg)	71.6 ± 15.9	72.5 ± 12.0	70.9 ± 10.4	0.92
BMI (kg/m^2^)	25.5 ± 4.3	26.4 ± 4.1	26.3 ± 4.0	0.75
SBP (mmHg)	127.0 ± 25.4^*^	134.0 ± 18.6	145.7 ± 7.0	0.03
DBP (mmHg)	74.3 ± 10.8^*^	73.7 ± 9.4 ^*^	83.7 ± 12.5	0.01
Biochemical profile				
Triglyceride (mg/dL)	150.4 ± 60.3	168.5 ± 98.2	179.1 ± 4.8	0.65
Total cholesterol (mg/dL)	189.8 ± 34.3	180.6 ± 31.7	178.4 ± 0.0	0.70
LDL (mg/dL)	115.5 ± 35	108.2 ± 29.7	95.1 ± 50.2	0.39
VLDL (mg/dL)	29.7 ± 12.6	33.8 ± 19.6	31.3 ± 23.3	0.74
HDL (mg/dL)	45.4 ± 10.1	38.6 ± 8.4	45.9 ± 25.6	0.14
T-cho/HDL	4.4 ± 1.3	4.9 ± 1.3 ^*^	3.2 ± 2.0	0.04
LDL/HDL	2.7 ± 1.1	2.9 ± 1.1	3.3 ± 3.7	0.74
Non-HDL (mg/dL)	144.4 ± 37.3	142.0 ± 30.3	116.2 ± 3.9	0.27
Fasting glucose (mg/dL)	121.2 ± 38.1	113.4 ± 77.1	93 ± 41.3	0.52
HbA1c (mg/dL)	6.4 ± 1.2	6.5 ± 1.5 ^*^	4.1 ± 2.7	0.03

Data are expressed as means ± standard deviations. *P* values are calculated from one-way analysis of variance

*Abbreviations*: HF, heart failure; *LVEF* left ventricular ejection fraction, *BMI* body mass index, *SBP* systolic blood pressure, *DBP* diastolic blood pressure, *DM* diabetes mellitus, *MI* prior myocardial infarction, *DCM* dilated cardiomyopathy, *HDL* high-density lipoprotein cholesterol, *T-cho* total cholesterol *VLDL* very low-density lipoprotein cholesterol, *LVL* low-density lipoprotein cholesterol, *HbA1c* glycated hemoglobin

^*^ *p* < 0.05 vs. controls

### CMR data

The CMR data of the study participants are summarized in Table 2. Compared with controls, our low LVEF group patients showed significant differences in all the functional CMR parameters, including lower LVEF (31.2 % vs. 70.9 %), higher LVEDV (169.5 mL vs. 95.3 mL), higher LVESV (120.8 mL vs. 28.2 mL), lower stroke volume (48.7 mL vs. 67.2 mL), lower cardiac output (3.7 L/min vs. 5.1 L/min), higher LV mass (122.2 vs. 94.4) and lower LVGFI (20.2 % vs. 46.2 %). Regarding our patients with normal LVEF as compared with controls, significant differences were observed in LVEF, LVGFI, LVESV and LV mass but not in LVEDV, stroke volume and cardiac output.

**Table 2:** CMR imaging data of the study participants

	HF patients with low LVEF	HF patients with normal LVEF	Control subjects	*p* value
	(*n* = 25)	(*n* = 23)	(*n* = 21)	
LV ejection fraction (%)	31.2 ± 12.3^*, **^	60.2 ± 8.3 ^*^	70.9 ± 7.6	<0.001
LV end-diastolic volume (mL)	169.5 ± 78.1^*^,^**^	124 ± 43.7 ^*^	95.3 ± 23.4	< 0.001
LV end-systolic volume (mL)	120.8 ± 70.7^*^,^**^	51.2 ± 24.0 ^*^	28.2 ± 11.5	< 0.001
Stroke volume (mL)	48.7 ± 19.4^*^,^**^	72.8 ± 22.4	67.2 ± 15.9	< 0.001
Cardiac output (L/min)	3.7 ± 1.5^*^,^**^	4.8 ± 1.5	5.1 ± 1.1	0.004
LV mass (g)	126.1 ± 52.9^*^	113.5 ± 34.8 ^*^	94.4 ± 23.2	0.032
LV global function index (%)	20.2 ± 9.0^*^,^**^	37.6 ± 6.7 ^*^	44.6 ± 6.1	< 0.001
LV concentricity	0.8 ± 0.3^*^,^**^	1.0 ± 0.4	1 ± 0.3	0.027

Data are expressed as means ± standard deviations. *P* values are calculated from one-way analysis of variance

*Abbreviations*: *HF* heart failure, *LV* left ventricle, *LVEF* left ventricular ejection fraction

^*^ *p* < 0.05 vs. controls; ^**^ *p* < 0.05 vs. HF patients with normal LVEF

### ^1^H-MRS data

**Fig. 3:**
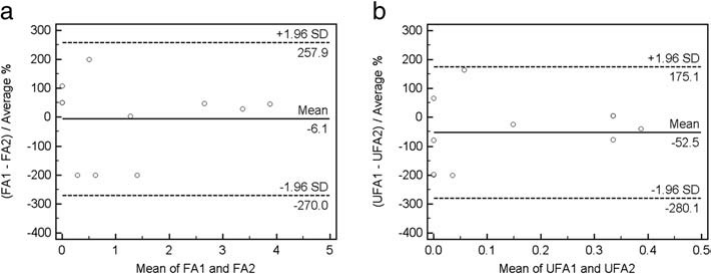
Bland-Altman plot. Reproducibility between two repeated ^1^H-MRS measures within a single CMR session in the first 10 subjects regarding (**a**) saturated fatty acid (FA) and (**b**) unsaturated fatty acid (UFA). Of note, two data points overlapped in the left lower corner on the UFA/W plot. SD, standard deviation

^1^H-MRS was found to be an accurate and reliable technique to quantify and discriminate between unsaturated fatty acids and fatty acids in the phantom study (Additional file 1: Figure S1). The Bland-Altman plots demonstrated that the differences for FA and UFA lay within the mean ± 1.96 SD in the first ten control subjects, suggesting that our results had an acceptable reproducibility [19] despite large confidence intervals (Fig. 3). The coefficient of variation (CV) of the individual spectra acquired without water suppression was 8 %, suggesting a robust and reproducible measurement. The ^1^H-MRS characteristics of the study participants are provided in Table 3. There were significant differences in terms of myocardial UFA/W content among the low LVEF group, the normal LVEF group, and controls (0.79 % vs. 0.21 % vs. 0.14 %, *p* = 0.02), but no in terms of TG/W and FA/W content. Moreover, the myocardial UFA/W content was significantly higher in the low LVEF group compared with the normal LVEF group (*p* = 0.03). The myocardial UFA/TG ratio was strongly associated with LV mass (*r* = 0.39, *p* < 0.001) and modestly related to LVEDV (*r* = 0.24, *p* = 0.039). We also identified negative correlations of myocardial FA/TG ratio with both LV mass (*r* = -0.39, *p* < 0.001) and LVEDV (*r* = -0.24, *p* = 0.039) (Table 4).

**Table 3:** CMR spectroscopy data of the study participants

	HF patients with low LVEF	HF patients with normal LVEF	Control subjects	*p* value
	(*n* = 25)	(*n* = 23)	(*n* = 21)	
TG/W (%)	1.72 ± 2.63	1.18 ± 1.82	1.53 ± 1.78	0.68
FA/W (%)	0.93 ± 2.13	0.97 ± 1.64	1.39 ± 1.67	0.66
UFA/W (%)	0.79 ± 1.38^*,**^	0.21 ± 0.28	0.14 ± 0.20	0.02
FA/TG (%)	0.53 ± 0.41	0.68 ± 0.40	0.71 ± 0.32	0.25
UFA/TG (%)	0.47 ± 0.41	0.32 ± 0.40	0.29 ± 0.32	0.25

Data are expressed as means ± standard deviation. *P* values are calculated from one-way analysis of variance

*Abbreviations*: *HF* heart failure, *LVEF* left ventricular ejection fraction, *TG* triglyceride; *FA* fatty acids, *UFA* unsaturated fatty acids, *W* water

^*^ *p* < 0.05 vs. controls; ^**^ *p* < 0.05 vs. HF patients with normal LVEF

**Table 4:** Correlations (Spearman’s rho) between the myocardial TG components and CMR features

Myocardial TG	MRI feature	Correlation coefficient (r)	*p* value
TG/W (%)	LVEF (%)	0.024	0.85
	LVEDV (mL)	−0.007	0.95
	LVESV (mL)	0.003	0.98
	LV stroke volume	0.026	0.83
	Cardiac output (L/min)	0.082	0.51
	LV mass (g)	−0.278	0.02
	LV global function index (%)	0.174	0.15
	LV concentricity	−0.280	0.02
FA/W (%)	LVEF (%)	0.161	0.19
	LVEDV (mL)	−0.194	0.11
	LVESV (mL)	−0.188	0.12
	LV stroke volume	−0.096	0.43
	Cardiac output (L/min)	−0.080	0.52
	LV mass (g)	−0.423	< 0.001
	LV global function index (%)	0.286	0.02
	LV concentricity	−0.146	0.23
UFA/W (%)	LVEF (%)	−0.177	0.15
	LVEDV (mL)	0.170	0.16
	LVESV (mL)	0.216	0.07
	LV stroke volume	0.034	0.78
	Cardiac output (L/min)	0.080	0.52
	LV mass (g)	0.006	0.96
	LV global function index (%)	−0.053	0.66
	LV concentricity	−0.253	0.04
FA/TG (%)	LVEF (%)	0.176	0.15
	LVEDV (mL)	−0.218	0.07
	LVESV (mL)	−0.233	0.05
	LV stroke volume	−0.101	0.41
	Cardiac output (L/min)	−0.128	0.30
	LV mass (g)	−0.345	0.004
	LV global function index (%)	0.206	0.09
	LV concentricity	−0.038	0.75
UFA/TG (%)	LVEF (%)	−0.178	0.14
	LVEDV (mL)	0.219	0.07
	LVESV (mL)	0.234	0.05
	LV stroke volume	0.092	0.45
	Cardiac output (L/min)	0.110	0.37
	LV mass (g)	0.366	0.002
	LV global function index (%)	−0.220	0.07
	LV concentricity	0.056	0.65

*Abbreviations*: *TG* triglyceride, *FA* fatty acids, *UFA* unsaturated fatty acids, *W* water, *LVEF* left ventricular ejection fraction, *LVEDV* left ventricular end-diastolic volume, *LVESV* left ventricular end-systolic volume, *LV* left ventricle

## Discussion

^1^H-MRS performed on 1.5 T MR scanner has been shown to be a reliable and reproducible imaging modality for the assessment of human myocardial TG content [11–13], while the high-field 3 T MR scanner, as used in this study, has significantly greater signal-to-noise ratio coupled with increased spectral resolution and has shown to be more accurate for in vivo quantification of myocardial fat fraction as compared to 1.5-Tesla [20]. ^1^H-MRS research study on formalin-fixed specimens of human hearts at various locations demonstrated that septal fat content is largely representative of myocardial TG deposition [21]. Herein, we applied ^1^H-MRS in the interventricular septum to assess the heart of a subgroup of HF patients 6 ~ 12 months after hospitalization on 3-T MR scanner. To the best of our knowledge, this is the first in vivo study specifically investigating the relation of different myocardial TG components and functional cardiac parameters on post-hospitalized HF patients with 3-T CMR plus ^1^H-MRS. Our main results indicated an association between the accumulation of myocardial TG components and the damaged heart. First, myocardial UFA content was significantly higher in our patients than in controls, even those showed good recovery from HF with their LVEF restoring to the normal range. Second, the myocardial UFA/TG ratio was positively correlated with LV mass and LVEDV. The reproducibility of our results was confirmed by Bland-Altman plots [19]. Taken together, the current study support the clinical utility of 3 T ^1^H-MRS for measuring myocardial TG components in patients who were hospitalized with acute HF.

In this study, our patients had significantly increased myocardial UFA content as compared with controls. Intriguingly, there was a stepwise decrease of myocardial UFA concentrations when comparing patients with low LVEF, normal LVEF, and controls (*p* = 0.02). The standard deviation of UFA/W was significantly larger in patients with low EF compared with values observed in patients with normal EF and controls (Table 3). This observation can be explained by the wider range of EF observed in patients with low EF, ultimately resulting in a higher standard deviation (12.3) than those observed in both subjects with normal EF (8.3) and controls (7.6, Table 2). The differences in the standard deviations are unlikely to be caused by a suboptimal UFA quantification. Accordingly, our phantom study clearly demonstrated the reproducibility of measuring and fitting UFA (Additional file: 1 Figure S1). The relationship of accumulation of myocardial TG in patients with cardiomyopathy has been previously reported, but with different results [14, 15, 22, 23]. Graner et al. [22] reported that myocardial TG content decreased in non-diabetic subjects with dilated cardiomyopathy, however, showed the opposite results in those with diabetic heart disease [14, 23]. Nakae et al. [15] found that myocardial TG may be related to the specific cause of disease rather than the severity of cardiac dysfunction. Nevertheless, notable finding from the current study is that myocardial UFA, instead of FA and TG, was increased in post-hospitalized HF patients and was related to the severity of LV systolic function.

LV mass and LVEDV of our patients were positively correlated with myocardial UFA/TG ratio but were negatively correlated with myocardial FA/TG ratio. Their correlations with TG/W were not significant. In a series of 15 healthy humans evaluated with 1.5 T 1H- MRS, Szczepaniak et al. [24] found that increased myocardial triglyceride content was accompanied by elevated LV mass. In another series with ten male endurance athletes and 15 healthy male controls evaluated with 1.5 T ^1^H- MRS, Sai et al. [25] showed that the myocardial TG content was significantly lower in the athlete group than in the control group, and was negatively correlated with LV mass and volume. We believe that the study participants selection and MRI scanner field strength may, at least, partly account to the discrepancies of our results from those previously reported. Of note, the averaged TG/W ratio found in our control group was 15.3 which fell between those of our stable HF (11.8) and unstable HF group (17.2). Accordingly, our control subjects had a mean body mass index of 26.3 kg/m^2^, being overweight according to the WHO definition. In addition, the TG/W ratio is characterized by a diurnal course, with higher levels in the morning than in the evening [9]. The impact of these potential confounders on the TG/W needs further scrutiny.

It has been reported that myocardial TG accumulation promotes the development of cardiac hypertrophy, ventricular dysfunction, and interstitial fibrosis [5]. Although an increased TG deposition in the pancreatic islets has been linked to non-insulin-dependent diabetes mellitus [26], the potential relationship between myocardial TG accumulation and diabetic heart disease remains unclear. An animal study has demonstrated that increased TG content within the myocardium contributes to the development of cardiac dysfunction through lipotoxic effects [27]. In patients with HF, accumulation of myocardial UFA may reflect a switch of the myocardial energy metabolism to the fetal transcriptional program with a reduced rate of β-oxidation, which is partially compensated by an increased of glucose utilization [28]. Moreover, Lahey et al. have shown that UFA are more potent activators of TG turnover than saturated FA in animal models. In decompensated HF, UFA may serve as a beneficial energy substrate versus FA by upregulating TG dynamics and nuclear receptor signaling [29]. Notwithstanding the scarce evidence, it has been alternatively suggested that myocardial TG accumulation may be protective against fatty acid-induced lipotoxicity by limiting the deposition of ceramides and diacylglycerols [30, 31]. Although our study did not provide the mechanisms how TG content mediates cardiac dysfunction, the in vivo identification of the associations of TG components, particularly UFA, with cardiac function using 3-T CMR in the post-treated failed heart is an important step forward.

The mean BMI of the entire study population (comprising both patients and controls) was 26.1 kg/m^2^, suggesting the presence of overweight according to the World Health Organization criteria [32]. Previous studies have linked the presence of cardiac steatosis with obesity and related metabolic diseases [10, 16, 17]. Notably, ectopic TG accumulation within and around the myocardium in moderately obese individuals has been associated with free fatty acid exposure, generalized ectopic fat excess, and peripheral vascular resistance [33]. However, we failed to identify significant associations between myocardial TG content and serum lipid levels. These results suggest that ^1^H-MRS measurements of myocardial TG content may provide complementary clinical information beyond serum lipid profile.

### Limitations

Several caveats of our study merit comment. First, our subgroup analysis is limited by the small sample size. Although myocardial UFA/TG was positively related with LV mass and LVEDV, these relationships were fairly weak (*r* = 0.39 and 0.24, respectively) and large confidence intervals for the spectral quantification were evident. Therefore, caution should be exercised in the interpretation of our findings. Future larger studies will be needed to establish whether myocardial UFA content may vary between HF patients with different etiologies. Second, ^1^H-MRS measurements of myocardial TG content were not validated by histological examination of biopsies, mainly because of ethical concerns. Finally, it is unclear whether the accumulation of myocardial TG results from increased uptake of fatty acids, higher *de novo* lipogenesis, or reduced lipid degradation. Further studies are required to clarify the pathophysiological mechanisms of myocardial TG accumulation in HF patients.

## Conclusions

Patients who were discharged after hospitalization for acute HF had increased myocardial UFA content as compared with controls. Furthermore, UFA was inversely related with LVEF, LV mass and, to a lesser extent, LVEDV. Our current report may stimulate further research aimed at measuring myocardial UFA content by ^1^H-MRS for predicting clinical outcomes.

## Additional file

Additional file 1: Figure S1. MRS of an oil phantom. Representative magnetic resonance spectrum of an oil phantom (85 % unsaturated fatty acid) at 3 T, with a 2 × 2 × 1-cm^3^ spectroscopic volume. ^1^H-MR spectra were analyzed using the LC Model software. Fatty acids (FA, lipid resonances δ 0.9, 1.3, and 1.6 ppm) and unsaturated fatty acids (UFA, lipid resonance δ 2.1 and 2.3, 2.8, 5.3 ppm). (TIFF 886 kb)

## Abbreviations

ANOVA: Analysis of variance
BMI: Body mass index
CMR: Cardiovascular magnetic resonance
CRLB: Cramer-Rao lower bound
DM: Diabetes mellitus
FA: Fatty acids
HF: Heart failure
^1^H-MRS: Proton magnetic resonance spectroscopy
LV: Left ventricle
LVEDV: Left ventricular end-diastolic volume
LVESV: Left ventricular end-systolic volume
LVGFI: Left ventricular global function index
LVSV: Left ventricular stroke volume
MR: Magnetic resonance
PRESS: Point resolved spectroscopy
SSFP: Steady-state free precession
TG: Triglyceride
UFA: Unsaturated fatty acids
W: Water
