# Supplementary figures and images for: Myocardial triglyceride content at 3 T cardiovascular magnetic resonance and left ventricular systolic function: a cross-sectional study in patients hospitalized with acute heart failure
Source: J Cardiovasc Magn Reson. 2016 Feb 5;18:9. doi: 10.1186/s12968-016-0228-3 (PMC4744377; doi:10.1186/s12968-016-0228-3)

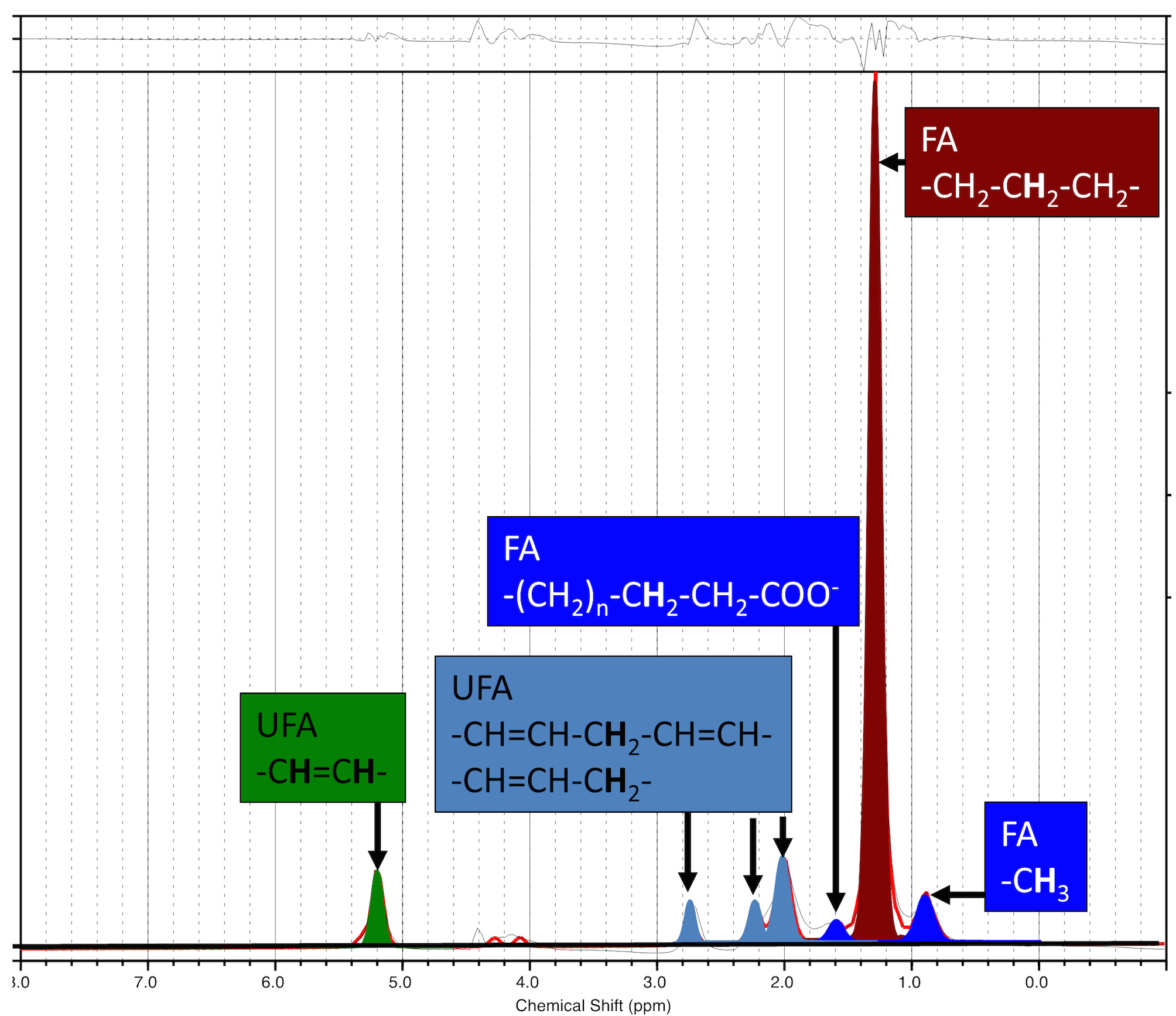

Supplement: Additional file 1: Figure S1. — MRS of an oil phantom. Representative magnetic resonance spectrum of an oil phantom (85 % unsaturated fatty acid) at 3 T, with a 2 × 2 × 1-cm3 spectroscopic volume. 1H-MR spectra were analyzed using the LC Model software. Fatty acids (FA, lipid resonances δ 0.9, 1.3, and 1.6 ppm) and unsaturated fatty acids (UFA, lipid resonance δ 2.1 and 2.3, 2.8, 5.3 ppm). (TIFF 886 kb) [file 12968_2016_228_MOESM1_ESM.tiff]
